# Supplementary material for: Injury prevents Ras mutant cell expansion in mosaic skin
Source: Nature. 2023 Jun 21;619(7968):167–75. doi: 10.1038/s41586-023-06198-y (PMC10322723; doi:10.1038/s41586-023-06198-y)
Supplement: Supplementary file 1 — Uncropped and unprocessed immunoblots of Fig. 5b (n = 3 mice for each condition), Extended Data Fig. 8b (n = 6 mice p-EGFR/EGFR, n = 3 mice p-ERK1/2/ERK1/2 and p-AKT/AKT) and Extended Data Fig. 10 a,b (n = 4 mice wild-type, n = 3 mice HrasG12V/+ mosaic, n = 3 mice HrasG12V/+ max). For statistical and densitometry analyses see figure legends and data sources. Membranes were cut around the molecular weight of interest before blotting. Molecular markers were acquired with colorimetric setting. Proteins were detected by western blotting, using the indicated antibodies and the red rectangles show the cropping location. [file 41586_2023_6198_MOESM1_ESM.pdf]

nature portfolio

https://doi.org/10.1038/s41586-023-06198-y

Supplementary information

Injury prevents Ras mutant cell expansion in mosaic skin

In the format provided by the authors and unedited

SI Figure 1

wild-type CTR / wild-type TREATED (x3 indipendent mice)

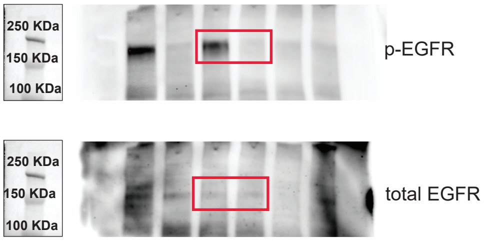

total EGFR was used as control for quatification
It was blot on the same membrane

wild-type CTR / wild-type TREATED (x3 indipendent mice)

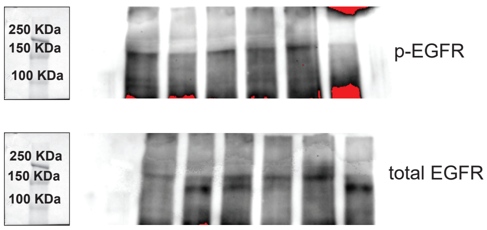

total EGFR was used as control for quatification
It was blot on the same membrane

wild-type CTR / wild-type TREATED (x3 indipendent mice)

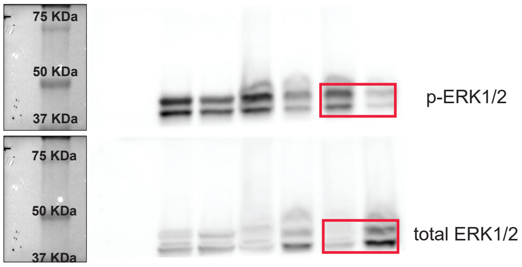

total ERK1/2 was used as control for quatification
It was blot on the same membrane

wild-type CTR / wild-type TREATED (x3 indipendent mice)

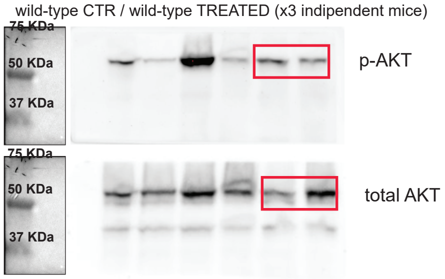

total ERK1/2 was used as control for quatification
It was blot on the same membrane

Raw data in Extended Fig. 8b

wild-type Uninjured/Injured (x3 indipendent mice)

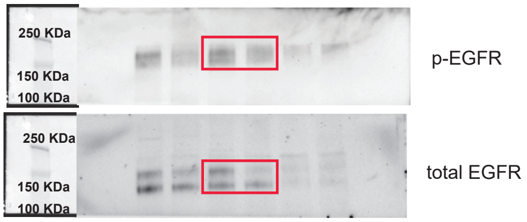

total EGFR was used as control for quatification of p-EGFR
It was blot on the same membrane

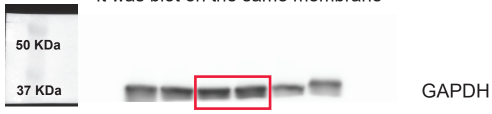

GAPDH was used as control for quatification of total EGFR
It was blot on the same membrane

Hras-MAX Uninjured/Injured (x3 indipendent mice)

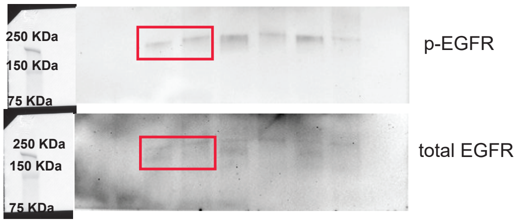

total EGFR was used as control for quatification of p-EGFR
It was blot on the same membrane

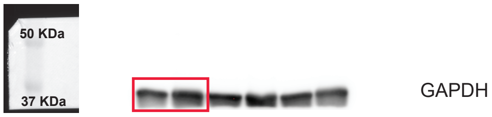

GAPDH was used as control for quatification of total EGFR
It was blot on the same membrane

Raw data in Fig. 5b

wild-type Uninjured/Injured(x1) - Hras-MAX Uninjured/Injured
(x3 indipendent mice)

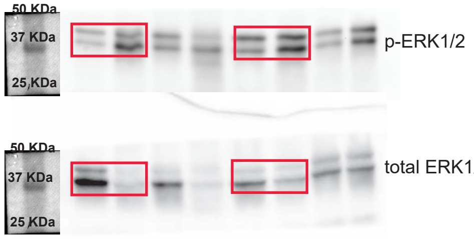

total ERK1/2 was used as control for quatification
It was blot on the same membrane

wild-type Uninjured/Injured(x1) - Hras-MOSAIC Uninjured/Injured
(x3 indipendent mice)

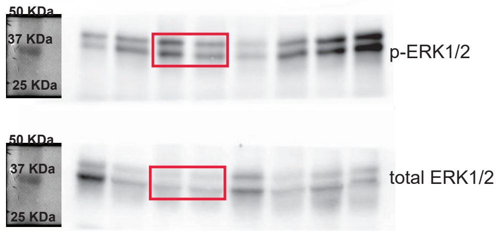

total ERK1/2 was used as control for quatification
It was blot on the same membrane

wild-type Uninjured/Injured Uninjured/Injured (x2) - p21null Uninjured
(x3 indipendent mice)

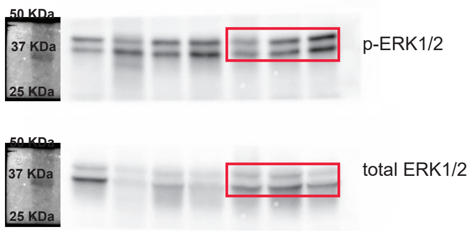

total ERK1/2 was used as control for quatification
It was blot on the same membrane

wild-type Uninjured/Injured(x1) - Hras-MAX Uninjured/Injured
(x3 indipendent mice)

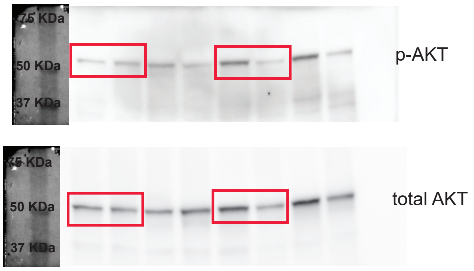

total AKT was used as control for quatification
It was blot on the same membrane

wild-type Uninjured/Injured(x1) - Hras-MOSAIC Uninjured/Injured
(x3 indipendent mice)

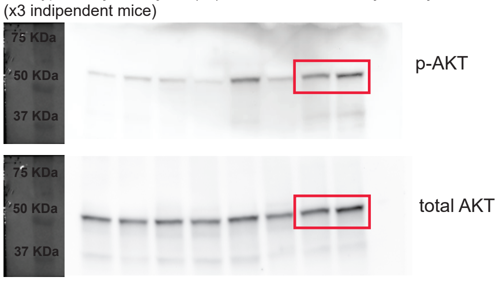

total AKT was used as control for quatification
It was blot on the same membrane

wild-type Uninjured/Injured Uninjured/Injured (x2) - p21null Uninjured
(x3 indipendent mice)

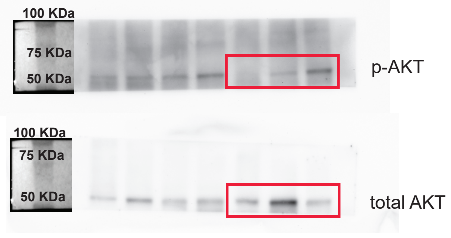

total AKT was used as control for quatification
It was blot on the same membrane

Raw data in Externded Fig.10a, b

SI Figure 1 – Uncropped and unprocessed immunoblots of Figure 5b (n=3 mice for each condition), Extended Figure Data Figure 8b (n=6 mice p-EGFR / EGFR, n=3 mice p-ERK1/2 / ERK1/2 and p-AKT / AKT) and Extended Data Figure 10 a, b (n=4 mice wild-type, n=3 mice HrasG12V/+-mosaic, n=3 mice HrasG12V/+-max). For statistical and densitometry analyses see figure legends and data sources. Membrane were cut around the molecular weight of interest before blotting. Molecular markers were acquired with colorimetric setting. Proteins were detected by western blotting, using the indicated antibodies and the red rectangles show the cropping location.
