# Supplementary material for: Injury prevents Ras mutant cell expansion in mosaic skin
Source: Nature. 2023 Jun 21;619(7968):167–75. doi: 10.1038/s41586-023-06198-y (PMC10322723; doi:10.1038/s41586-023-06198-y)
Supplement: Supplementary file 2 — Reporting Summary [file 41586_2023_6198_MOESM2_ESM.pdf]

nature portfolio

Corresponding author(s): Valentina Greco Maria Kasper

Last updated by author(s): Mar 31, 2023

Reporting Summary

Nature Portfolio wishes to improve the reproducibility of the work that we publish. This form provides structure for consistency and transparency in reporting. For further information on Nature Portfolio policies, see our Editorial Policies and the Editorial Policy Checklist.

Statistics

For all statistical analyses, confirm that the following items are present in the figure legend, table legend, main text, or Methods section.

n/a	Confirmed	
☐	☒	The exact sample size (*n*) for each experimental group/condition, given as a discrete number and unit of measurement
☐	☒	A statement on whether measurements were taken from distinct samples or whether the same sample was measured repeatedly
☐	☒	The statistical test(s) used AND whether they are one- or two-sided<br>*Only common tests should be described solely by name; describe more complex techniques in the Methods section.*
☒	☐	A description of all covariates tested
☐	☒	A description of any assumptions or corrections, such as tests of normality and adjustment for multiple comparisons
☐	☒	A full description of the statistical parameters including central tendency (e.g. means) or other basic estimates (e.g. regression coefficient) AND variation (e.g. standard deviation) or associated estimates of uncertainty (e.g. confidence intervals)
☐	☒	For null hypothesis testing, the test statistic (e.g. *F*, *t*, *r*) with confidence intervals, effect sizes, degrees of freedom and *P* value noted<br>*Give P values as exact values whenever suitable.*
☒	☐	For Bayesian analysis, information on the choice of priors and Markov chain Monte Carlo settings
☒	☐	For hierarchical and complex designs, identification of the appropriate level for tests and full reporting of outcomes
☒	☐	Estimates of effect sizes (e.g. Cohen's *d*, Pearson's *r*), indicating how they were calculated

Our web collection on statistics for biologists contains articles on many of the points above.

Software and code

Policy information about availability of computer code

Data collection	Image stacks were acquired with a LaVision TriM Scope II (LaVision Biotec) laser scanning microscope equipped with a tunable Two-photon Chameleon Vision II (Coherent) Ti:Sapphire laser and Two-photon Chameleon Discovery (Coherent) Ti:Sapphire laser. ImSpector v7.5.2 (LaVision Biotec) for 3D image acquisition.<br>Image stacks of epidermal preparation immunolabeled against phosphoERK1/2 and phalloidin were acquired with confocal microscope Zeiss LSM 980 with Software ZEN (blue edition).<br>To prepare the single-cell library, the cellular suspensions were counted and diluted to a final concentration of 1200 cells/μl in PBS/0.04% BSA and then loaded on a Chromium Controller to generate single-cell gel bead emulsions, targeting 3′. Single-cell 3′ RNA-seq libraries were generated according to the manufacturer’s instructions (Chromium Single Cell 3′ Reagent v3 Chemistry Kit, 10X Genomics, Inc.). Libraries were sequenced to an average depth of ?20.000 reads per cell on an Illumina Novaseq 6000 system.
Data analysis	Statistical analyses were performed using Prism (version 9) as indicated in the figure legends. Raw two-photon image stacks were analyzed in ImageJ (1.53c, NIH Image) or IMARIS (version 9.9.1, Oxford Instruments). To quantify the thickness of the skin epithelium, we used IMARIS and MatLab (version R2018a). To quantify the mean fluorescence intensity of the p21 signal within each individual nucleus and background we used IMARIS. Single cell RNA-sequencing analysis was performed in Scanpy (1.6-1.9), SoupX (https://github.com/constantAmateur/SoupX), Seurat (3 - https://satijalab.org/seurat/index.html), bbknn (1.4.1), DoubletFinder package (https://github.com/chris-mcginnis-ucsf/DoubletFinder), scikit-learn (0.24.2), miloR (1.2.0) and GSEAPY package (v 0.12). Further details are provided in methods and analysis notebooks are uploaded to github: (https://github.com/kasperlab/Gallini_et_al_2023_Nature).

For manuscripts utilizing custom algorithms or software that are central to the research but not yet described in published literature, software must be made available to editors and reviewers. We strongly encourage code deposition in a community repository (e.g. GitHub). See the Nature Portfolio guidelines for submitting code & software for further information.

Data

Policy information about availability of data

All manuscripts must include a data availability statement. This statement should provide the following information, where applicable:

- Accession codes, unique identifiers, or web links for publicly available datasets
- A description of any restrictions on data availability
- For clinical datasets or third party data, please ensure that the statement adheres to our policy

All data from this study are available from the authors on request. The raw data files of the scRNA-seq analyses reported in the manuscript are uploaded to Gene Expression Omnibus (GSE195892). Previously published scRNA-seq data that were used for reference are available under accession codes GSE152044, GSE129218, and GSE67602. Annotated and analysed sequencing data have been deposited in Zenodo: https://doi.org/10.5281/zenodo.7768108 and analysis notebooks are uploaded to GitHub: https://github.com/kasperlab/Gallini_et_al_2023_Nature.

Human research participants

Policy information about studies involving human research participants and Sex and Gender in Research.

Reporting on sex and gender	N/A
Population characteristics	N/A
Recruitment	N/A
Ethics oversight	N/A

Note that full information on the approval of the study protocol must also be provided in the manuscript.

Field-specific reporting

Please select the one below that is the best fit for your research. If you are not sure, read the appropriate sections before making your selection.

☒ Life sciences ☐ Behavioural & social sciences ☐ Ecological, evolutionary & environmental sciences

For a reference copy of the document with all sections, see nature.com/documents/nr-reporting-summary-flat.pdf

Life sciences study design

All studies must disclose on these points even when the disclosure is negative.

Sample size	No statistical method were used to pre-determined sample sizes but our sample sizes are similar to those reported in previous publications (Rompolas, Mesa et al., 2016, Brown, Pineda et al., 2017, Mesa, Kawaguchu, Cockburn et al., 2018, Joost et al., 2016, Joost, Annusver et al., 2020, Cockburn Annusver et al., 2022).
Data exclusions	No data were excluded from the analysis
Replication	All the experiments were performed in at least 3 biologically independent replicates. All replicates reported in the manuscript are biological replicates. All attempts at replication of the results were successful.
Randomization	Mouse models were chosen based on correct genotypes: K14CreER/FR-HrasG12V/tdTomato/K14H2B-GFP; K14CreER/tdTomato/K14H2B-GFP; K14CreER/LSL-KrasG12D/tdTomato/K14H2B-GFP; K14CreER/FR-HrasG12V/p21null/tdTomato/K14H2B-GFP; K14CreER; K14rtTA; FR-HrasG12V/+; LSL-tdTomato; TRE-EGFR-DN; K14H2B-GFP.<br>Mice were always induced with tamoxifen (Sigma T5648-5G) at 19 days after birth and wound was induce at 21 days after birth. Each experiment contained animals from at least 2 different litters.
Blinding	The investigator were not blinded . Blinding was not possible as the same investigator processed the animals and analyzed the data.

Reporting for specific materials, systems and methods

We require information from authors about some types of materials, experimental systems and methods used in many studies. Here, indicate whether each material, system or method listed is relevant to your study. If you are not sure if a list item applies to your research, read the appropriate section before selecting a response.

Materials & experimental systems

n/a	Involved in the study
☐	☒ Antibodies
☒	☐ Eukaryotic cell lines
☒	☐ Palaeontology and archaeology
☐	☒ Animals and other organisms
☒	☐ Clinical data
☒	☐ Dual use research of concern

Methods

n/a	Involved in the study
☒	☐ ChIP-seq
☒	☐ Flow cytometry
☒	☐ MRI-based neuroimaging

Antibodies

Antibodies used

For Immunofluorescence the following antibodies were used:
Primary antibodies: active-Caspase3 (AF835-R&D Systems, Inc.) 1:300, phospho-Histone3 (06-570-Millipore) 1:300, Keratin6A (905701-BioLegend) 1:500, p21/Cdkn1a (ab188224-Abcam) 1:50; Phospho-p44/42 MAPK(Erk1/2) (4310-Cell Signaling) 1:300, Alexa Fluor™ 647 Phalloidin (A22287-Thermofisher) 1:200 and Keratin10 (03-GP-K10-ARP) 1:200 diluted in blocking buffer. Secondary antibodies: Alexa Fluor 633, A-21071-Invitrogen-Goat anti-Rabbit IgG (H+L) Secondary Antibody, Alexa Fluor 633, A-21105-Invitrogen-Goat anti-Guinea Pig IgG (H+L) Highly Cross-Adsorbed Secondary Antibody, Alexa Fluor™ 488, A-21206, Donkey anti-Rabbit IgG (H+L) Highly Cross-Adsorbed, and Alexa Fluor 568, A10042-Donkey anti-Rabbit IgG (H+L) Highly Cross-Adsorbed Secondary Antibody) were diluted 1:300.
For Western Blot the following rabbit primary antibodies were used at the given concentrations: phospho-p44/42 MAPK (ERK1/2) (Thr202/Tyr204) (1:500, Cell Signaling-9101), p44/42 MAPK (ERK1/2) (1:500, Cell Signaling-4695), phosphoEGFR (Tyr1068) (1:100, Cell Signaling-2234), EGFR (1:100 Cell Signaling-4267 - Extended Data Figure 8b), EGFR (1:100, Cell Signaling-2232 - Figure 5b), phosphoAKT (Ser473) (1:200, Cell Signaling-4060), AKT (1:200, Cell Signaling-9262) and GAPDH (14C10) (1:500 - Cell Signaling-2118).
An anti-rabbit IgG HRP (1:500, Cell Signaling-7074) secondary was used.

Validation

Antibodies validation information can be found on manufacturers' website. We used protocols and recommendations of the manufacturer on validated species.

Primary Antibodies:
1. active-Caspase3 (AF835-R&D Systems, Inc - https://www.rndsystems.com/products/human-mouse-active-caspase-3-antibody_af835)
2. phospho-Histone3 (06-570-Millipore - https://www.emdmillipore.com/US/en/product/Anti-phospho-Histone-H3-Ser10-Antibody-Mitosis-Marker,MM_NF-06-570)
3. Keratin6A (905701-BioLegend - https://www.biolegend.com/en-us/search-results/purified-anti-mouse-keratin-6a-antibody-11459)
4. p21/Cdkn1a (ab188224-Abcam - https://www.abcam.com/products/primary-antibodies/p21-antibody-epr18021-ab188224.html)
5. Phospho-p44/42 MAPK (Erk1/2) (Thr202/Tyr2 - 04) (D13.14.4E) XP® Rabbit mAb #4370 - https://www.cellsignal.com/products/primary-antibodies/phospho-p44-42-mapk-erk1-2-thr202-tyr204-d13-14-4e-xp-rabbit-mab/4370
6. Keratin10 (03-GP-K10-ARPv - https://www.arp1.com/anti-keratin-k10-polyclonal-antibody-serum-03-gp-pp2.htmlv / Cockburn, Annusver et al., 2022)
7. phospho-p44/42 MAPK (ERK1/2) (Thr202/Tyr204) (Cell Signaling-9101 - https://www.cellsignal.com/products/primary-antibodies/phospho-p44-42-mapk-erk1-2-thr202-tyr204-antibody/9101)
8. p44/42 MAPK (ERK1/2) (Cell Signaling-4695 - https://www.cellsignal.com/products/primary-antibodies/p44-42-mapk-erk1-2-137f5-rabbit-mab/4695)
9. phosphoEGFR (Tyr1068) (Cell Signaling-2234 - https://www.cellsignal.com/products/primary-antibodies/phospho-egf-receptor-tyr1068-antibody/2234)
10. EGFR (Cell Signaling-2232 - https://www.cellsignal.com/products/primary-antibodies/egf-receptor-antibody/2232)
11. EGFR (Cell Signaling-4267 -https://www.cellsignal.com/products/primary-antibodies/egf-receptor-d38b1-xp-rabbit-mab/4267)
12. phosphoAKT (Ser473) (Cell Signaling-4060 - https://www.cellsignal.com/products/primary-antibodies/phospho-akt-ser473-d9e-xp-rabbit-mab/4060)
13. AKT (Cell Signaling-9262 - https://www.cellsignal.com/products/primary-antibodies/akt-antibody/9272)
14. GAPDH (14C10) (Cell Signalling-2118 - https://www.cellsignal.com/products/primary-antibodies/gapdh-14c10-rabbit-mab/2118)

Secondary Antibodies:
1. Alexa Fluor 633, A-21105-Invitrogen-Goat anti-Guinea Pig IgG (H+L) Highly Cross-Adsorbed Secondary Antibody - https://www.thermofisher.com/antibody/product/Goat-anti-Guinea-Pig-IgG-H-L-Highly-Cross-Adsorbed-Secondary-Antibody-Polyclonal/A-21105
2. Alexa Fluor 633, A-21071-Invitrogen-Goat anti-Rabbit IgG (H+L) Secondary Antibody (https://www.thermofisher.com/antibody/product/Goat-anti-Rabbit-IgG-H-L-Highly-Cross-Adsorbed-Secondary-Antibody-Polyclonal/A-21071)
3. Alexa Fluor™ 647 Phalloidin (A22287-Thermofisher - https://www.thermofisher.com/order/catalog/product/A22287v)
4. Alexa Fluor™ 488, A-21206, Donkey anti-Rabbit IgG (H+L) Highly Cross-Adsorbed (https://www.thermofisher.com/antibody/product/Donkey-anti-Rabbit-IgG-H-L-Highly-Cross-Adsorbed-Secondary-Antibody-Polyclonal/A-21206)
5. Alexa Fluor 568, A10042-Donkey anti-Rabbit IgG (H+L) Highly Cross-Adsorbed Secondary Antibody (https://www.thermofisher.com/antibody/product/Donkey-anti-Rabbit-IgG-H-L-Highly-Cross-Adsorbed-Secondary-Antibody-Polyclonal/A10042)
6. An anti-rabbit IgG HRP (1:1000, Cell Signaling-7074 - https://www.cellsignal.com/products/secondary-antibodies/anti-rabbit-igg-hrp-linked-antibody/7074v)

Animals and other research organisms

Policy information about studies involving animals; ARRIVE guidelines recommended for reporting animal research, and Sex and Gender in Research

Laboratory animals	An outbred mouse strain background (CD1) from post-natal day 19 to post-natal day 49 was used in this study. K14CreER (Vasioukhin et al., 1999) and "Flox and Replace"-HrasG12V/+ (Chen et al., 2009), constitutive p21 (Cdkn1a) loss of function (Deng et al., 1995; JAX stock #016565), LoxSTOPLox-tdTomato (Madisen et al., 2010; JAX stock #007909), K14H2B-GFP (Tumbar et al.,2004), K14rtTA (Nguyen et al., 2006; (JAX stock #008099)), TRE-EGFR-DN (Roh et al., 2001; (JAX stock #010575)) and and LoxSTOPLox-KrasG12D/+ (Kackson et al., 2001). All the animals used were grown in mixed albino background (CD1) to allow two-photon imaging experiments.<br>To induce genetic recombination CreER-driven all mice were subjected to an intraperitoneal tamoxifen injection at 19 days after birth. At post-natal day 21, mice were anaesthetized by intraperitoneal injection of ketamine and xylazine cocktail mix (100mg kg and 10mg kg, respectively in phosphate-buffered saline). Once the anesthetized mouse did not physically respond to a noxious stimulus, a punch biopsy was performed using a 4-mm-diameter punch biopsy tool on the dorsal side of a mouse ear or in back skin. For recovery from the wound procedure Meloxicam(Metacam® Loxicom®) was administered via subcutaneous injection (0.3 mg Kg).<br>For in vivo imaging, mice were anaesthetized by intraperitoneal injection of ketamine and xylazine cocktail mix (100mg kg and 10mg kg, respectively in phosphate-buffered saline) and then anesthesia was maintained throughout the course of the experiment with the delivery of vaporized isoflurane by a nose cone.<br>K14CreER (Vasioukhin et al., 1999) and "Flox and Replace"-HrasG12V/+ (Chen et al., 2009), constitutive p21 (Cdkn1a) loss of function (Deng et al., 1995; JAX stock #016565), LoxSTOPLox-tdTomato (Madisen et al., 2010; JAX stock #007909), K14H2B-GFP (Tumbar et al.,2004), K14rtTA (Nguyen et al., 2006; (JAX stock #008099)), TRE-EGFR-DN (Roh et al., 2001; (JAX stock #010575)) and and LoxSTOPLox-KrasG12D/+ (Kackson et al., 2001). All the animals used were grown in mixed albino background to allow two-photon imaging experiments.<br>To induce genetic recombination CreER-driven all mice were subjected to an intraperitoneal tamoxifen injection (Sigma T5648-5G in corn oil) at 19 days after birth.<br>To induce rtTA-driven induction of EGFR-DN, mice were administered 2% of Doxycycline (Sigma D9891) and 2% sucrose (Sigma S9378) in drinking water. All time courses began 6 days post-tamoxifen injection. Gefitinib (ZD1839-Selleckchem) was resuspended in water with 0.5% (w/v) methylcellulose and 0.2% (v/v) Tween-80 (vehicle) and was administered orally (200 mg/kg body weight) starting 2 days before wound induction until 14 days post-wound induction.<br>At post-natal day 21, mice were anaesthetized by intraperitoneal injection of ketamine and xylazine cocktail mix (100mg kg and 10 mg kg, respectively in phosphate-buffered saline). Once the anesthetized mouse did not physically respond to a noxious stimulus, a punch biopsy was performed using a 4-mm-diameter punch biopsy tool (Integra™ Miltex™ Standard Biopsy Punches) on the dorsal side of a mouse ear or in back skin.<br>For in vivo imaging, mice were anaesthetized by intraperitoneal injection of ketamine and xylazine cocktail mix (100mg kg and 10mg kg, respectively in phosphate-buffered saline) and then anesthesia was maintained throughout the course of the experiment with the delivery of vaporized isoflurane by a nose cone.<br><br>Housing condition: five mice per cage<br>Dark/light cycle: light from 7 AM to 7 PM<br>Ambient temperature and humidity: 68-79°F and humidity 30-70%.
Wild animals	This study did not involve wild animals.
Reporting on sex	Sex-specific differences were minimized by including both male and female animals in the replicates.
Field-collected samples	The study did not involve samples collected from the field.
Ethics oversight	All procedures involving animal subjects were performed under the approval of the Institutional Animal Care and Use Committee (IACUC) of the Yale School of Medicine. The mice were sacrificed if tumor reached 1cm3, not allowed by IACUC, or if mice presented signs of distress or weight loss. The tumor size limit was not exceeded in any of the experiments.

Note that full information on the approval of the study protocol must also be provided in the manuscript.
